# The Law of Cure, the Guide

**Published:** 1882-12

**Authors:** Carroll Dunham


### THE LAW OF CURE: THE GUIDE.

It has been taught by some authors, and believed by many members of the homœopathic faith, that Aconite and Belladonna—except as intercurrents during the congestive stage of heat—are useless in the treatment of intermittent fever. But the law of cure, as enunciated by Hahnemann, knows no such narrow restriction and is not bound by the *ipse dixit* of individual opinion.

If Aconite or Belladonna covers the totality of the patient's symptoms, it will as certainly cure this fever as any other remedy. They are comparatively rarely indicated, but will effectually do their work when called for. The characteristic symptoms of the remedy must always be the guide.—CARROLL DUNHAM.

